# Qualitative study of comprehension of heritability in genomics studies among the Yoruba in Nigeria

**DOI:** 10.1186/s12910-020-00567-2

**Published:** 2020-12-09

**Authors:** Rasheed O. Taiwo, John Ipadeola, Temilola Yusuf, Faith Fagbohunlu, Gbemisola Jenfa, Sally N. Adebamowo, Clement A. Adebamowo, the Indigene Study as part of the H3Africa Consortium

**Affiliations:** 1 Division of Research Ethics, Center for Bioethics and Research, Ibadan, Nigeria; 2 Department of Epidemiology and Public Health, Greenebaum Comprehensive Cancer Center, University of Maryland School of Medicine, Baltimore, MD USA; 3 Institute of Human Virology, Abuja, Nigeria; 4 Institute of Human Virology Building, School of Medicine, University of Maryland, 725 West Lombard Street, Baltimore, MD 21201 USA

**Keywords:** Linguistic concepts, Heritability, Comprehension, Informed consent, Genomics studies, FGD, KII, Yoruba indigenous communities

## Abstract

**Background:**

With growth of genomics research in Africa, concern has arisen about comprehension and adequacy of informed consent given the highly technical terms used in this field. We therefore decided to study whether there are linguistic and cultural concepts used to communicate heritability of characters, traits and diseases in an indigenous African population.

**Methods:**

We conducted Focus Group Discussions among 115 participants stratified by sex, age and socio-economic status and Key Informant Interviews among 25 stakeholders and Key Opinion Leaders among Yoruba living in Ibadan, Nigeria. We used Atlas-ti v.8.3.17 software to analyze the data, using thematic approach.

**Results:**

The study participants identified several linguistic and cultural concepts including words, proverbs, and aphorisms that are used to describe heritable characters, traits and diseases in their local dialect. These included words that can be appropriated to describe dominant and recessive traits, variations in penetrance and dilution of strength of heritable characteristics by time and inter-marriage. They also suggested that these traits are transmitted by “blood”, and specific partner’s blood may be stronger than the other regardless of sex.

**Conclusions:**

Indigenous Yoruba populations have words and linguistic concepts that describe the heritability of characters, traits and diseases which can be appropriated to improve comprehension and adequacy of informed consent in genomics research. Our methods are openly available and can be used by genomic researchers in other African communities.

## Background

The number of genomics research projects in Sub-Saharan Africa (SSA) is growing thanks to initiatives such as the Human Heredity and Health in Africa (H3Africa) Consortium. The H3Africa Consortium is a joint initiative of the African Society of Human Genetics (AfSHG), United States’ National Institutes of Health (NIH) and Wellcome Trust designed to “harness genomic technologies towards improving health in Africa” while contributing to global health [1–15]. H3Africa also supports development of genomics research infrastructure and builds African capacity for genomics research through provision of training in diverse fields [16–29]. The outcome of this initiative is an increase in the number of research projects focused on the genomic bases of diseases, phenotypic adaptations, pharmacogenomics and population histories in Africa [1–15, 30, 31]. These studies combine modern genomics technology with unparalleled and detailed phenotypic variations [30–34].

The implementation of genomics studies in SSA has been accompanied by significant ethical concerns including questions about the quality and adequacy of informed consent, community engagement, dignitary harms, risk of stigmatization and discrimination arising from the outcome of genomics research [35–37]. In particular, the presentation of technical terms used in genomics research during the informed consent process and the adequacy of comprehension of such terms by research participants in low-resource countries is an important ethical and scientific question [38].

Research participants need to adequately comprehend informed consent before it can be considered valid [39]. Some studies have suggested there is poor understanding of genomics among indigenous communities due to low levels of literacy [36, 46–50]. While some components of the informed consent process are similar across all research studies and words that most people are familiar with are often used, genomics research utilizes several technical terms or describes technical processes that community members may not be aware of and this increases the likelihood of inadequate comprehension. Many of the terms used in genomics research are neologisms and they are used to express complex biomedical processes that may not yet be well known in popular culture. Therefore local language equivalents for these terms do not exist in many societies, particularly those in low and middle income countries (LMIC) [40]. Nevertheless, these societies may not be entirely ignorant of the concepts expressed by these genomics terms.

In this study, we examined the linguistic and cultural concepts that are used to describe heritability of characters, traits and diseases by Yoruba living in Nigeria. Heritability is a measure of how well differences in people’s genes account for differences in their traits and diseases while a heritable trait is an offspring’s trait that resembles the parents’ corresponding trait more than it resembles the same trait in a random individual in the population [41, 42]. Although the literacy level, particularly for genomics concepts in Nigeria, like in most LMIC, is low, we hypothesized that Yoruba participants in genomics research are able to comprehend these concepts because of availability of words, axioms, aphorisms, and sayings in their language that are used to communicate heritability. Further, we hypothesized that identifying and incorporating such words and concepts into the informed consent process will improve participant’s comprehension of informed consent and their engagement in genomics research. The “Indigenous linguistic and cultural concepts of heritability and comprehension of genomics research in Nigeria” (INDIGENE study) was designed to use qualitative research methods to elicit these words and use them to design “enhanced informed consent” forms that are compared with standard informed consent in a randomized trial. In this paper, we report the results of the qualitative study among the Yoruba in Nigeria.

## Methods

### Study population and sample

We purposively sampled 140 participants who are at least 18 years old, had not previously participated in any genomics research project, of Yoruba ethnicity, speak Yoruba language and reside in Ibadan, a predominantly Yoruba metropolis in South-Western Nigeria. They were recruited through personal contacts and series of community engagement activities where we explained the study and its objectives. We used Focus Group Discussions (FGD) and Key Informant Interviews (KII) to elicit participants’ knowledge of the linguistic and cultural concepts used to describe heritability of traits and diseases in Yoruba culture.

#### Focus Group Discussions (FGD)

For the FGD, we conducted 12 FGD, each with 10 invited participants. We had a very high response rate and all eligible individuals who were invited, agreed to participate in the study. However, we excluded one woman due to uncorrected hearing impairment. Four male participants were unable to attend the FGDs at scheduled times, leaving 115 participants in the FGDs. Two of the focus groups had male participants only, four groups had females only and six groups had mixed male and female participants. We balanced each FGD on basis of religion, age and socio-economic status.

We conducted the FGDs at suitable, private venues in Ibadan, each by one researcher and a note taker. The FGDs were led by JI, a male researcher, TY, FF and GJ, female researchers. All the researchers have extensive experience in conducting FGDs. We used FGD guides that were carefully edited to avoid pre-loading with concepts that may inadvertently influence participants’ answers. We conducted the FGDs in Yoruba language and each of them lasted for one hour on average. We recorded the discussions, translated them to English language and transcribed them. JI, TY, FF and GJ verified each of the transcriptions against the audio recordings and notes taken during the FGDs. JI, TY, FF, GJ, ROT, SNA and CAA reviewed and analyzed all the audio recordings.

#### Key Informant Interview (KII)

After the FGDs, we invited 25 individuals who did not participate in the group discussions and are considered key opinion leaders and custodians of the community’s folk and oral traditions, to participate in key informant interviews. The participants were recruited through contacts in the community and guidance from participants in our FGDs. No individual refused our invitation to participate in the interview.

JI, TY, FF and GJ conducted the KIIs using interview guides and took supplementary notes. We used scenario-based discussion guides that we updated with results from our earlier FGDs in order to elicit more in-depth responses, and by using follow-up questions, prompts and probes. Similar to the FGDs, we used KII guides that we edited to avoid pre-loading with concepts that may inadvertently influence participants’ answers. We employed reflective methods during the interviews and noted participants’ observations and subjective experiences. We conducted the interviews in homes, offices and other locations that were most convenient for the participants. The KIIs were conducted in either English or Yoruba depending on the preference of the participants. Each KII lasted for an average of one hour. The interviews conducted in Yoruba were translated to English and transcribed. The transcribed documents were reviewed and analyzed by JI, TY, FF, GJ, ROT, SNA and CAA.

The FGD and KII were overseen by CAA (male, physician, bioethicist, and epidemiologist) and SNA (female, physician and epidemiologist) who have experience and publication record in use of qualitative research methods [43–46].

### Data analysis

After verification with the supplementary notes taken during the sessions, we imported the transcriptions of the FGDs and KIIs into Atlas-ti version 8.3.17 (Atlas.ti Scientific Software Development, GmbH Berlin, Germany) for analysis. We used thematic approach to analyze the FGDs and KIIs [47, 48]. Each researcher, JI, TY, FF, GJ and ROT reviewed the transcribed recordings and used an iterative process to generate an initial level of codes which were evaluated by CAA and SNA for secondary and tertiary level codings. We grouped related codes into families and created ‘networks’ of hierarchical themes. We analyzed each network for emerging themes and sub-themes and kept memos containing contextual information during this process. All the authors reviewed the codes, resolved coding discrepancies and agreed on a final set of codes. We used the consolidated criteria for reporting qualitative research to describe our results [47, 48].

## Results

### Study participants

There were 140 participants in this study, 69 of whom were men (52 in FGD and 17 in KII) while 71 were women (63 in FGD and 8 in KII). The mean (SD) age of participants was 61.0 (15.2) years. Less than 50% of the participants in the FGD had formal education and were literate. This is lower than the literacy rate (62.9%) of the general population where the study was conducted. The KII participants were all literate and had formal education. Our participants were traders, artisans, local government officials, administrators, religious leaders, local chiefs, head teachers and community leaders by occupation.

### Respondents’ view on Yoruba’s belief on heritability of characters, traits and diseases from parents/progenitors

Most of the study participants indicated that the Yoruba believe that some traits and characters such as physical features (height, color of the skin, eyes, etc.) and diseases are heritable from parents. Specific attributes such as facies, skin color and height are expected of family offsprings else the child is considered a bastard. For instance, some FGD participants expressed the belief that “*a n fi awo jo iran eni*”, meaning a person’s skin traits or complexion is heritable from his family gene. Other examples of heritable traits given by our participants include albinism (*afin*), dwarfism (*rara*), etc.

### Characters, traits and diseases commonly attributed to heritability by Yoruba

Respondents mentioned illnesses they considered heritable and their indigenous names. These include: hypertension (*eje ruru*), sickle cell disease (*arunmolegun*), post-partum depression (*abisinwin)*, mental illnesses *(arun opolo)*, migraine (*oritulu*), hernia or hydroceles (*ipa*), some types of fever (*iba*) and hemorrhoids (*tapa*). They also identified excessive anger (*inubibi*), patience *(suuru*), stubbornness *(agidi*), and stealing (*ole-jija)* as some characters and traits that can be inherited. For example, one of the KII respondents affirmed that:

“Physical features like the shape of the ear (*bi eti se ri*), skin color (*awo*); characters like thuggery (*iwa ipanle*) and alcoholism (*oti amupara*); as well as illnesses like epilepsy (*warapa*), ulcer (*ogbe inu*), etc. are examples of the traits and diseases that are transferred from generation to generation.”

### Words, phrases or proverbs used to describe heritable traits.

Virtually all respondents indicated that there are words, phrases, proverbs and other expressions in Yoruba that are commonly used to indicate or demonstrate their understanding of heritability. Examples of such expressions are:

*“omo erin jogun ola”* (the child has inherited the family’s attributes);

“*bomo o jo sokoto, yio jo kijipa, eni o bini la jo*” (the child has to display some of the characters or traits of his parents);

“*ere sisa la fi bi eshin*” (horses inherit the ability to race);

“*owu ti iya gbon lomo o ran*” (children must take after their parents);

“*omo ajanaku kan ki ya ara, ohun ti eya bi leya jo*” (a warrior’s child will always be a warrior).

These common expressions confirm an appreciation of the concepts of heritability of traits and the availability of words, phrases and proverbs to convey them in Yoruba language.

Our participants also displayed an awareness of the heritability of physical traits through an expectation that children should physically resemble members of their family. In the absence of such traits and attributes, they may say:

“*niboni won tigbe omo eyi wa”* (meaning the progenitor of this child is questionable – either because of the presence or absence of specific family traits, characters or illnesses)*;*

*imi o jo eni to su u (meaning a child (behavior) does not resemble his parents);*

“*omo-aale*’’ (a bastard).

Some of the respondents emphasized that “these” words show that they recognize or expect transmission of characters, traits or diseases to offsprings. For example:

*“o jo baba re bi imumu”* (meaning the child is a replica of his father);

*omo ti ekun ba bi, ekun nijo* (a lion’s offspring has characteristic traits of a lion, implying that like begets like), and so on.

In support of this, other respondents emphasized that:

*eku diran oje, ise baba re ni i se* (meaning that the character traits that a child exhibits are inherited from his father (and known with his ancestors).

### Understanding of transmission of traits

Most participants in the FGD and KII believed that traits, characteristics and diseases can be passed down from one generation to another. They believed that the factor responsible for this is in the blood and it is exchanged during sexual intercourse. They also reported that some traits can be transmitted from mother to child through breast feeding. Furthermore, they indicated an understanding that traits or diseases may be present in previous generations like grandparents and appear in the children but not the parents. Their belief is that:

“*inu eje ibi lo ma n wa*” (the essence responsible for transmission of traits and diseases resides in a component of blood).

Most of the respondents said that Yoruba believe that a child will necessarily inherit certain traits from either or both parents because he “shares the same blood with them” and because male and female sex gametes are released during coitus. If a child does not share any trait with his parents or any relative, this is viewed seriously and considered highly suspicious of infidelity on the part of the mother.

### Understanding of the concept of dominant traits.

The respondents expressed their understanding of the concept of dominant traits by saying:

“*eje kan le mu ju ikeji lo*”,

which means, some “blood” may be dominant over the other in the context of transmissibility of heritable traits/characteristics, and the “stronger blood” could be that of the mother or the father. They gave examples such as couples having children who are all of a particular sex, of children predominantly exhibiting traits or characteristics found in only one parent or in one parent’s family, regardless of the sex of the child. For instance, when a child overly manifest characteristic traits of his mother, they say:

*omo ja ifun iya re je* (meaning that a child takes after his mother in character or behavior).

In discussing the concept of dominant traits, one FGD participant said that:

“if the blood of the father is stronger, the child will resemble the father; and if the mother’s blood is stronger, the child will resemble the mother.”

Thereby showing that these concepts are not gendered when used in these particular contexts.

### Perception of heritability of sickle cell trait and disease.

We asked our discussants about heritability of sickle cell disease (SCD), a common and well known disease in the Yoruba community. Many of the discussants identified SCD, a disease associated with recurrent episodes of excrutiating bone pain, hence the local name *“arunmolegun (bone crushing disease of children)”* as a heritable disease. They recognized that the severity of the disease varies from individuals to individuals, and that this variation in severity may reflect variations in the strength of the influence of the causative and associated factors in individuals. Respondents also recognized that SCD may manifest in some children and not in others within the same family which they attributed to randomness.

### Secular variation in heritable traits and diseases

Some participants in the FGD reported that the strength (penetration) of some traits and diseases may be “diluted” through intermarriages and over time. One of the participants said:

“Traits or diseases that run in families become less and less potent in future generations until reaching zero level.”

A female KII participant said that:

“The severity of heritable disease such as SCD reduces across generations within a family.”

## Discussion

Our study showed that there are words and concepts in Yoruba language that can be appropriated to communicate about heritability of characters, traits and diseases and serve as a foundation for informed consent discussions in genomics research in this community. These words, phrases, proverbs, idioms and aphorisms are used to express understanding of heritability and allude to its role in specific situations. Participants used these words to express an understanding of ideas in genomics such as dominant traits, recessive traits and their relationship to health and diseases, such as sickle cell disease. They also appreciated the possibility of dilution of strength of inherited characters, traits and diseases over time in a population.

Our participants mentioned several characters such as alcoholism, traits like height, body build, skin color, eye color, body shape and facies, and diseases such as sickle cell disease, epilepsy, mental illness and hypertension as having heritable components. This belief in heritability of characters, traits and diseases is the basis for the Yoruba cultural practice of engaging “*alarina”* (a liaison/go-between/investigator) to examine the family histories of intending couples before marriage is contracted among the Yoruba. With few dissenting voices, most of our participants believed that these traits and illnesses are transmitted through the blood (*eje*). They indicated that they mean that “the heritability essence is in the blood” and not the physicality of “blood”.

Previous studies on the understanding of genomics research among indigenous communities, suggested that the low levels of literacy in these communities may limit their ability to comprehend technical terms used in genomics research [36, 49–53]. The results of our study suggest that incorporation of the indigenous words and concepts that are used to discuss heritability of characters, traits and diseases may enhance comprehensibility of consent forms and improve quality of informed consent. Our study participants demonstrated a rich vocabulary of such words, phrases and proverbs.

Comprehension of genomics research requires more than a literal translation of consent forms into local languages in order to improve ‘readability’ or ensure provision of all ‘required information’ [54–56]. In order to ensure comprehension of scientific concepts, especially when specific words or expressions do not exist in local languages, mere transliteration of the technical concepts may be insufficient [38]. Drawing from existing cultural and linguistic concepts allow interpretation of complex concepts like genomics into words and languages that are comprehensible to indigenous research participants and reduce the influence of limited availability of scientific and technical terms on comprehension of consent in genomics research in Africa [57].

Communication of genomics to research participants in developing countries has typically relied on a transmission model of communication to explain informed consent. This model assumes ‘stable senders and receivers and unambiguous messages’ [58]. Hence, research participants in LMIC are often labelled ‘incompetent’ or ‘non-compliant’ when they fail to understand the language used to explain specific research methods such as genomics [59]. However, communication theory has shown that perception and communication are dependent on the ‘frames and references’ of the receiver and sender of the message [60]. Our study reinforces that knowledge gained from communication is subject to discourse and the familiarity of ‘frames and references’ to the receiver of the message. Thus identifying and integrating relevant “frames and references” is a useful way of explaining complex concepts such as those encountered in genomic research to participants [61].

Integration of cultural frames and references to convey meaning and ideas is common in other spheres of communication in Africa. African writers who publish in English language often integrate local linguistic and cultural concepts into their prose. An example is discussed in Walter’s description of Wole Soyinka’s ‘*The Trials of Brother Jero’*. In this work, Soyinka—a Nigerian Nobel laureate in literature—applies ‘code-switching and code-mixing’ using linguistic concepts in Yoruba, English and West African pidgin English [62]. Hence, the play is a heterogeneous mix of diverse linguistic and cultural concepts that help contextualize and convey the message within the play more poignantly to the African reader. A similar approach to communicating informed consent will improve comprehension of informed consent and empower African research participants and their communities in genomics research [63].

To facilitate informed consent in genomic research in this environment, we have created a publicly available database of the words and concepts that we identified in the course of this study (https://indigenestudy.bioethicscenter.net). We invite other stakeholders in ethics of genomics research to work with us in building up this database and using it to improve comprehension of informed consent in genomics research. We have also posted our methodology for elicitation of indigenous words and concepts for genomics research online for researchers who wish to replicate similar studies in their environment. These resources will be useful to other researchers as they develop tools for genomics research studies.

Our study has a few limitations. Because ours is a qualitative study, its generalizability is limited. Nevertheless, it provides information on which to base future quantitative and experimental studies which are more generalizable. Our sample size was small but is similar to that of other qualitative studies. We acknowledge that some words, concepts, adages and proverbs in indigenous languages can be ethically and scientifically problematic so we do not advocate wholesale uncritical appropriations. We ensured that our study participants were balanced by religion, age, sex, and socio-economic status. In future studies, we will randomize participants in ongoing breast cancer genomics research among the Yoruba to the current informed consent and an enhanced informed consent that incorporates local words and concepts based on insights gained from this study.

## Conclusions

Our study showed that there are indigenous words and concepts used to communicate about heritability of characters, traits and diseases among the Yoruba in Nigeria. These can be used to enhance comprehension of informed consent and improve the adequacy of consent for genomics research in this community. Similar approaches can be used to improve informed consent for genomics research in other African societies and we have created an open source database of words and our methodology to enhance this process.

## Data Availability

We created a publicly available database of the words and concepts that were identified in the course of this study (https://indigenestudy.bioethicscenter.net). We also created an online resource on the same website describing our methodology for elicitation of indigenous words and concepts for genomics research.
